# Supplementary material for: Detailed molecular and epigenetic characterization of the pig IPEC-J2 and chicken SL-29 cell lines
Source: iScience. 2023 Feb 20;26(3):106252. doi: 10.1016/j.isci.2023.106252 (PMC10018572; doi:10.1016/j.isci.2023.106252)
Supplement: Data S2. Complete homer output for identified motifs in Chicken SL-29, related to Tables 5 and 6 — Homer motif analysis results for histone modifications H3K4me1, H3K4me3, H3K27ac, enhancers, and ATAC-seq of chicken SL-29 cell line. Parameters for possible false positives is as mentioned earlier for S5. [file mmc3.zip › Data_S2/S6/Chicken_SL_29/peak_files_H3K4me3/homerResults.html]

peak\_files\_H3K4me3/ - Homer de novo Motif Results


# Homer *de novo* Motif Results (peak\_files\_H3K4me3/)

Known Motif Enrichment Results  
Gene Ontology Enrichment Results  
If Homer is having trouble matching a motif to a known motif, try copy/pasting the matrix file into
STAMP  
More information on motif finding results: HOMER
| Description of Results
| Tips
  
Total target sequences = 17161  
Total background sequences = 32688  
\* - possible false positive  

|  |  |  |  |  |  |  |  |  |
| --- | --- | --- | --- | --- | --- | --- | --- | --- |
| Rank | Motif | P-value | log P-pvalue | % of Targets | % of Background | STD(Bg STD) | Best Match/Details | Motif File |
| 1 | T C A G C T G A C G T A C G T A T C A G G C A T C T A G C G T A C G T A C T G A T A C G G A C T C T G A | 1e-232 | -5.355e+02 | 3.40% | 0.61% | 81.4bp (99.9bp) | IRF1(IRF)/PBMC-IRF1-ChIP-Seq(GSE43036)/Homer(0.974) More Information \| Similar Motifs Found | motif file (matrix) |
| 2 | A G C T A G T C A C T G C A G T C G A T C G T A C A G T A G T C | 1e-137 | -3.172e+02 | 58.27% | 48.73% | 85.1bp (101.5bp) | OVOL2/MA1545.1/Jaspar(0.751) More Information \| Similar Motifs Found | motif file (matrix) |
| 3 | T A C G A C T G A C G T C T A G G T C A A C T G A G C T T C A G | 1e-105 | -2.420e+02 | 28.26% | 21.20% | 83.3bp (92.3bp) | MSANTD3/MA1523.1/Jaspar(0.848) More Information \| Similar Motifs Found | motif file (matrix) |
| 4 | C T A G G T A C T G A C C A T G G T A C T G C A G T C A G C A T | 1e-87 | -2.008e+02 | 49.32% | 41.80% | 87.7bp (95.8bp) | Zfp57(Zf)/H1-ZFP57.HA-ChIP-Seq(GSE115387)/Homer(0.833) More Information \| Similar Motifs Found | motif file (matrix) |
| 5 | A G T C A G T C A C G T T A C G C G T A A T G C A T C G A C T G | 1e-76 | -1.766e+02 | 55.89% | 48.80% | 84.2bp (94.1bp) | AP-2alpha(AP2)/Hela-AP2alpha-ChIP-Seq(GSE31477)/Homer(0.734) More Information \| Similar Motifs Found | motif file (matrix) |
| 6 | T C A G C T A G A C T G A T G C G A T C A G C T T A C G T C G A T A C G A T C G | 1e-63 | -1.469e+02 | 24.38% | 19.13% | 78.7bp (95.8bp) | ZNF711(Zf)/SHSY5Y-ZNF711-ChIP-Seq(GSE20673)/Homer(0.865) More Information \| Similar Motifs Found | motif file (matrix) |
| 7 | T C G A G T C A T G C A T G A C G A T C G A T C A T G C T A C G | 1e-48 | -1.116e+02 | 50.97% | 45.37% | 84.2bp (95.1bp) | REL/MA0101.1/Jaspar(0.702) More Information \| Similar Motifs Found | motif file (matrix) |
| 8 | A T G C T A G C A C T G A C G T G C A T A T C G C G A T C T G A | 1e-47 | -1.094e+02 | 20.67% | 16.42% | 87.0bp (96.4bp) | PB0194.1\_Zbtb12\_2/Jaspar(0.636) More Information \| Similar Motifs Found | motif file (matrix) |
| 9 | A G T C A T C G G T A C G A C T A C T G C G T A A C T G T C A G C A G T A T C G | 1e-38 | -8.964e+01 | 7.75% | 5.35% | 85.0bp (91.0bp) | TFAP2C(var.2)/MA0814.2/Jaspar(0.661) More Information \| Similar Motifs Found | motif file (matrix) |
| 10 | C A G T A C T G G A T C G C A T G A T C C A T G G A C T A G C T A C G T A G T C | 1e-37 | -8.603e+01 | 30.88% | 26.48% | 84.5bp (94.2bp) | PB0137.1\_Irf3\_2/Jaspar(0.602) More Information \| Similar Motifs Found | motif file (matrix) |
| 11 | T G C A T C G A T G C A T G C A G C T A T C A G T A G C T C G A C T A G T G A C | 1e-31 | -7.343e+01 | 26.38% | 22.53% | 84.9bp (96.7bp) | ZNF384/MA1125.1/Jaspar(0.616) More Information \| Similar Motifs Found | motif file (matrix) |
| 12 | A G T C A C G T A T G C G A T C A G T C A C G T A C T G C G T A C G T A A C T G A G T C C G T A A T G C | 1e-25 | -5.774e+01 | 0.15% | 0.01% | 63.5bp (61.1bp) | TFAP2A/MA0003.4/Jaspar(0.654) More Information \| Similar Motifs Found | motif file (matrix) |
| 13 | A T G C A T G C A G C T T A C G C G A T C A T G A C T G T A C G T A C G A G C T A G T C A C G T T A C G | 1e-24 | -5.739e+01 | 5.99% | 4.29% | 79.9bp (88.3bp) | ZNF692(Zf)/HEK293-ZNF692.GFP-ChIP-Seq(GSE58341)/Homer(0.704) More Information \| Similar Motifs Found | motif file (matrix) |
| 14 | T G C A A G C T A G C T G A T C G T A C A G C T G T C A A G T C A T G C C T A G | 1e-23 | -5.306e+01 | 7.35% | 5.52% | 86.4bp (91.5bp) | TEAD(TEA)/Fibroblast-PU.1-ChIP-Seq(Unpublished)/Homer(0.682) More Information \| Similar Motifs Found | motif file (matrix) |
| 15 | A C T G C G T A C T A G C T A G A C G T A C T G A T C G A C G T A T G C A C G T A C T G A C G T A C T G | 1e-22 | -5.144e+01 | 0.13% | 0.01% | 72.5bp (68.3bp) | ZNF684/MA1600.1/Jaspar(0.635) More Information \| Similar Motifs Found | motif file (matrix) |
| 16 | A G C T A G C T A G C T A G C T A G C T A G C T A G C T A C T G G A T C G A T C G A T C G A T C G A T C | 1e-19 | -4.464e+01 | 8.44% | 6.64% | 86.2bp (105.9bp) | ZNF384/MA1125.1/Jaspar(0.723) More Information \| Similar Motifs Found | motif file (matrix) |
| 17 | A T G C A G C T C G T A C T G A A G T C A G T C A G T C A C T G | 1e-19 | -4.438e+01 | 9.91% | 7.97% | 84.4bp (97.0bp) | PB0024.1\_Gcm1\_1/Jaspar(0.737) More Information \| Similar Motifs Found | motif file (matrix) |
| 18 | C G T A A C G T A C T G A G T C C G T A A G T C A C T G A G T C C G T A A G C T C G T A G T A C C G T A | 1e-18 | -4.262e+01 | 0.13% | 0.01% | 75.9bp (57.5bp) | Arnt:Ahr(bHLH)/MCF7-Arnt-ChIP-Seq(Lo\_et\_al.)/Homer(0.675) More Information \| Similar Motifs Found | motif file (matrix) |
| 19 | A C T G A C T G C G T A A C G T C T G A C G A T C T A G A C T G T C A G C G T A A C G T A C G T C T G A | 1e-15 | -3.471e+01 | 0.12% | 0.01% | 80.5bp (129.1bp) | OTX2/MA0712.2/Jaspar(0.674) More Information \| Similar Motifs Found | motif file (matrix) |
| 20 | C T A G C T A G T A C G T C G A C A G T A T G C C A T G C A T G T A C G T C G A C A G T A C G T C T G A | 1e-14 | -3.363e+01 | 0.10% | 0.01% | 82.0bp (65.8bp) | PITX2/MA1547.1/Jaspar(0.679) More Information \| Similar Motifs Found | motif file (matrix) |
| 21 | G T C A G A C T A G C T A T G C A G T C A G C T T G A C C A T G C A T G G C T A | 1e-13 | -3.160e+01 | 5.00% | 3.84% | 85.4bp (96.9bp) | STAT6(Stat)/Macrophage-Stat6-ChIP-Seq(GSE38377)/Homer(0.705) More Information \| Similar Motifs Found | motif file (matrix) |
| 22 | G T A C A C T G C T G A A G C T C G A T A G T C A G T C A G T C | 1e-13 | -3.100e+01 | 8.44% | 6.94% | 82.2bp (91.4bp) | RELB/MA1117.1/Jaspar(0.759) More Information \| Similar Motifs Found | motif file (matrix) |
| 23 \* | A C G T C T A G G T C A C G A T A G T C C G A T A G C T A C T G A G T C C T A G | 1e-11 | -2.762e+01 | 0.36% | 0.13% | 84.4bp (88.1bp) | ESRRA/MA0592.3/Jaspar(0.683) More Information \| Similar Motifs Found | motif file (matrix) |
| 24 \* | A G C T C G T A C G A T C T A G A C T G C T G A G T C A A T C G A C T G A G T C C G T A A C T G A C G T | 1e-11 | -2.757e+01 | 0.12% | 0.02% | 66.9bp (42.9bp) | ZNF528(Zf)/HEK293-ZNF528.GFP-ChIP-Seq(GSE58341)/Homer(0.580) More Information \| Similar Motifs Found | motif file (matrix) |
| 25 \* | A C G T A C T G A C T G A C T G A C T G A G T C C G T A C T A G C T G A A C T G A G T C A T G C A G T C | 1e-11 | -2.545e+01 | 0.08% | 0.01% | 73.7bp (57.5bp) | PPARE(NR),DR1/3T3L1-Pparg-ChIP-Seq(GSE13511)/Homer(0.737) More Information \| Similar Motifs Found | motif file (matrix) |
| 26 \* | C T A G A G C T C T G A C G A T T G C A A C G T C T G A A G C T C T G A C G A T | 1e-9 | -2.257e+01 | 1.18% | 0.73% | 78.6bp (88.2bp) | PB0198.1\_Zfp128\_2/Jaspar(0.833) More Information \| Similar Motifs Found | motif file (matrix) |
| 27 \* | G A T C A G C T C T G A G A C T C G T A T C A G C T G A C T A G A G T C A G C T A G T C A G C T C T G A | 1e-9 | -2.246e+01 | 0.22% | 0.06% | 86.7bp (78.8bp) | PRDM14(Zf)/H1-PRDM14-ChIP-Seq(GSE22767)/Homer(0.602) More Information \| Similar Motifs Found | motif file (matrix) |
| 28 \* | G A T C A C G T C A T G G C A T C T G A A C G T C G A T A G T C C T A G C G T A | 1e-9 | -2.146e+01 | 0.10% | 0.02% | 95.4bp (0.0bp) | PB0171.1\_Sox18\_2/Jaspar(0.601) More Information \| Similar Motifs Found | motif file (matrix) |
| 29 \* | A C G T A C T G G C A T A C T G C T A G C T A G C T A G C G A T A C G T A C T G A C T G C T A G A C T G | 1e-8 | -2.061e+01 | 0.28% | 0.10% | 88.9bp (69.6bp) | Zfp281(Zf)/ES-Zfp281-ChIP-Seq(GSE81042)/Homer(0.746) More Information \| Similar Motifs Found | motif file (matrix) |
| 30 \* | C G T A C G T A A C G T A C G T A G T C C G T A A G T C A G T C A G T C A G T C | 1e-2 | -6.429e+00 | 0.05% | 0.02% | 72.2bp (48.5bp) | PB0030.1\_Hnf4a\_1/Jaspar(0.743) More Information \| Similar Motifs Found | motif file (matrix) |
